# Comparative effects of Er:YAG laser, and EDTA, MTAD, and QMix irrigants on adhesion of stem cells from the apical papilla to dentin: A scanning electron microscopic study 

**DOI:** 10.4317/jced.59129

**Published:** 2022-04-01

**Authors:** Afsaneh Rahmati, Hamed Karkehabadi, Golriz Rostami, Manoochehr Karami, Rezvan Najafi, Loghman Rezaei-Soufi

**Affiliations:** 1 Endodontics department, School of dentistry, Hamadan University of Medical Science, Hamadan, Iran; 2 Endodontist, Laser Research Center of Dentistry, Dentistry Research Institute, Tehran University of Medical Science, Tehran, Iran; 3 Dental Research Center, Department of Operative Dentistry, School of dentistry, Hamadan University of Medical Science, Hamadan, Iran; 4 Department of Epidemiology, School of Public Health & Saftey, Shahid Beheshti University of Medical Science, Tehran, Iran; 5 Department of Epidemiology, School of Public Health, Hamadan University of Medical Sciences, Hamadan, Iran; 6 Department of Medical Molecular & Genetics, Faculty of Medicine, Hamadan University of Medical Sciences, Hamadan, Iran

## Abstract

**Background:**

Dentin conditioning can affect the adhesion of stem cells in endodontic regenerative treatments. This study aimed to assess the effects of the most commonly used endodontic irrigants, namely, ethylenediaminetetraacetic acid (EDTA), MTAD, and QMix in comparison with Er:YAG laser (as a novel modality for root canal disinfection) on the adhesion of stem cells from the apical papilla (SCAPs) to dentin.

**Material and Methods:**

Forty dentin specimens were prepared and subjected to different treatments in 5 groups (n=8) of control, irrigation with EDTA for 1 minute, irrigation with MTAD for 5 minutes, irrigation with QMix for 5 minutes, and Er:YAG laser irradiation. SCAPs were isolated from third molar tooth buds that two-thirds of their roots had formed. The cells were cultured on dentin specimens for 3 days and were counted using scanning electron microscopy (SEM).

**Results:**

MTAD resulted in significantly lower adhesion of cells to dentin compared with other groups (*P*<0.05). All other modalities induced cell adhesion with no significant difference with each other (*P*>0.05).

**Conclusions:**

Despite many favorable properties, MTAD cannot serve as an optimal irrigant in endodontic regenerative procedures since it inhibits the adhesion of SCAPs to dentin and impairs an important step in tissue engineering.

** Key words:**Endodontic Regeneration, Er-YAG laser, MTAD, QMix, EDTA, SCAP, stem cell adhesion.

## Introduction

Endodontic regenerative procedures are biologically-based processes designed for predicTable replacement of the lost or injured tooth structures such as dentin, root, or dentin-pulp complex. Ideally, the lost tooth structures should be replaced with tissues of the same origin in order to reinstate their normal physiological functions (1,2).

In an optimal, successful regeneration treatment, it is imperative to induce the adhesion and attachment of dental pulp stem cells or other types of stem cells to dentin. Presence of smear layer on the root canal walls may inhibit or impair the adhesion of stem cells (which have the potential to promote regenerative processes) to dentin. Smear layer is a 1-5-µm increment of denatured debris created by instrumentation, which is composed of dentin chips, odontoblastic processes, non-specific mineral and organic compounds, and microorganisms (2,3). The available literature largely agrees on the necessity of smear layer removal from the instrumented root canal walls. Smear layer removal has advantages such as enhancing the root canal seal by the root filling materials, better penetration of irrigating solutions, and subsequently more efficient root canal disinfection and release of growth factors affecting adhesion, migration, and differentiation of stem cells (2,4). However, the effects of irrigants used for smear layer removal on adhesion, migration, and differentiation of stem cells have not been clearly elucidated.

QMix is a novel endodontic irrigant composed of polyamino carboxylic acid as chelator [17% ethylenediaminetetraacetic acid (EDTA)], biguanide as an antimicrobial agent (2% chlorhexidine), deionized water, and surfactant. It can effectively eliminate the smear layer and expose the dentinal tubules. In addition to smear layer removal, it has optimal antimicrobial properties for elimination of resistant bacteria such as Enterococcus faecalis (5). Evidence shows that application of QMix after irrigation with 5.25% sodium hypochlorite is as efficacious as the application of 17% EDTA (6,7). Also, QMix is more biocompatible than NaOCl and EDTA (6,8).

EDTA serves as a chelator; it binds to calcium ions and releases them from the crystalline structure of hydroxyapatite. Thus, it degrades the mineral phase of dentin. A high number of growth factors are present in demineralized extracellular matrix such as transforming growth factor-B1, fibroblast growth factor-2, bone morphogenetic protein-2, platelet-derived growth factor, placental growth factor, epidermal growth factor, and angiogenic factors such as vascular endothelial growth factor. The key role of these growth factors in promotion of tissue regeneration has been well documented (9).

MTAD was first introduced by Torabinejad and Johnson in Loma Linda University in 2003. It is composed of an aqueous phase containing 3% doxycycline as a broad-spectrum antibiotic, 4.25% citric acid as a chelator and demineralizing agent, detergent, and 0.5% polysorbate 80 (tween 80) as surfactant. Doxycycline hydrochloride has been used instead of free-base doxycycline monohydrate in the composition of MTAD due to its higher water solubility (10). MTAD is supplied as a two-component mixture, and is technically effective for smear layer removal and has optimal biocompatibility (10,11).

Laser irradiation has also been used for smear layer removal and dentin conditioning. Nd:YAG and CO2 lasers can eliminate the smear layer, uncover the dentinal tubules, and expose the collagen fibers with minimal change in the diameter of the tubules (12). A recent study revealed that root surface irradiated by Er:YAG laser had high biocompatibility for the attachment of periodontal ligament fibroblasts (13). They suggested that application of Er:YAG laser is a promising technique for dentin conditioning in regenerative treatments. All the aforementioned irrigants as well as Er:YAG laser irradiation can alter the dentin surface and may enhance or impede regenerative treatments. To date, limited studies are available regarding the effect of irrigating solutions and laser therapy on the adhesion of stem cells to dentin as a key step in success of tissue engineering treatments. Thus, this study aimed to assess the effects of the most commonly used endodontic irrigants namely EDTA, MTAD, and QMix, and Er:YAG laser irradiation (as a highly popular novel technology) on the adhesion of stem cells from the apical papilla (SCAPs) to root dentin for revascularization (regenerative) procedures.

## Material and Methods

-*Treatment of specimens*.

The study protocol was approved by the ethics committee of Hamadan University of Medical Sciences (IR.UMSHA.REC.1396.685). Twenty single-rooted extracted teeth of patients presenting to the School of Dentistry of Hamadan University of Medical Sciences were used for this study. The teeth had been extracted for purposes not related to this study.

Immediately after extraction, the teeth were gently scrubbed with a sterile brush to eliminate blood, saliva and the residual soft tissue. They were then rinsed with sterile saline. Each tooth was sectioned at two points with a diamond disc and low-speed hand-piece under sterile water coolant. The first section was made at 5 mm distance from the cementoenamel junction, and the second section was made at 2 mm distance from the first section. For the purpose of standardization of specimens in terms of tubular density and quality of root dentin at different distances from the cementoenamel junction, only the segment in the middle was used for this study. Next, the specimens were sectioned along their longitudinal axis in buccolingual direction to expose the pulpal wall. Two specimens were prepared from each tooth. To prevent possible contamination from the pulp, a cylindrical bur attached to a low-speed hand-piece was held parallel to the longitudinal axis of the root to remove 0.5 mm from the pulpal wall (in contact with the pulp tissue). All 40 specimens were immersed in 5.25% sodium hypochlorite and rinsed with sterile saline for 1 minute. The specimens were then randomized into five groups as follows:

(I) Control group: The specimens in this group were not irrigated with any irrigant and were not subjected to Er:YAG laser irradiation either.

(II) EDTA group: The specimens were immersed in 17% EDTA for 1 minute.

(III) MTAD group: The specimens were immersed in MTAD for 5 minutes.

(IV) QMix group: The specimens were immersed in QMix for 5 minutes.

(V) Laser group: The specimens were subjected to Er:YAG laser irradiation with 2.94 µm wavelength, 25 mJ energy per pulse, 15 Hz frequency, and 20 seconds of pulse duration. Laser was irradiated perpendicular to the specimen surface (13,14).

-*Culture of SCAPs*.

The apical papilla tissue was collected from the third molars of young adults between 16 to 18 years. A minimum of two-thirds of the third molar roots had formed. Written informed consent was obtained from the donors. The collected tissue was diced into equal-size pieces that were digested by using 3 mg/cc collagenase type I and 4 mg/cc dispase at 37°C for 30 minutes. The tissue suspension was filtered through a filter with 70 µm diameter and homogenized. The cells were then cultured in culture plates with 25 cm diameter in modified Eagle’s medium containing 15% to 20% fetal bovine serum, ascorbic acid 2-phosphate, penicillin, streptomycin, and Fungizone, and incubated in a humid incubator at 37°C and 5% CO2. After reaching 80% confluence, the cells were passaged and the cell content was assigned to three flasks (15). Third-passage cells were used for the experiment. Next, flow cytometric analysis was performed to ensure the mesenchymal origin of the cultured cells. Stem cells with mesenchymal origin have specific surface markers such as CD44,Stro-1,CD146,CD90,CD105. Presence of surface markers such as CD34 and CD45 indicate hematopoietic and leukocytic origin, respectively, which were not required for the present study (15).

-*Cell culture on dentin specimens*.

Prior to the application of cells on dentin specimens, the cells were balanced by immersion in 10% fetal bovine serum for 2 hours. The cells were detached by sterile trypsin-EDTA and after dilution by 105 cells/cc, they were cultured on dentin specimens for 3 days. Finally, they were rinsed with Dulbecco’s phosphate buffered saline and fixed with 4% glutaraldehyde (16).

-*Preparation of specimens for scanning electron microscopy (SEM)*.

**Figure 1:**
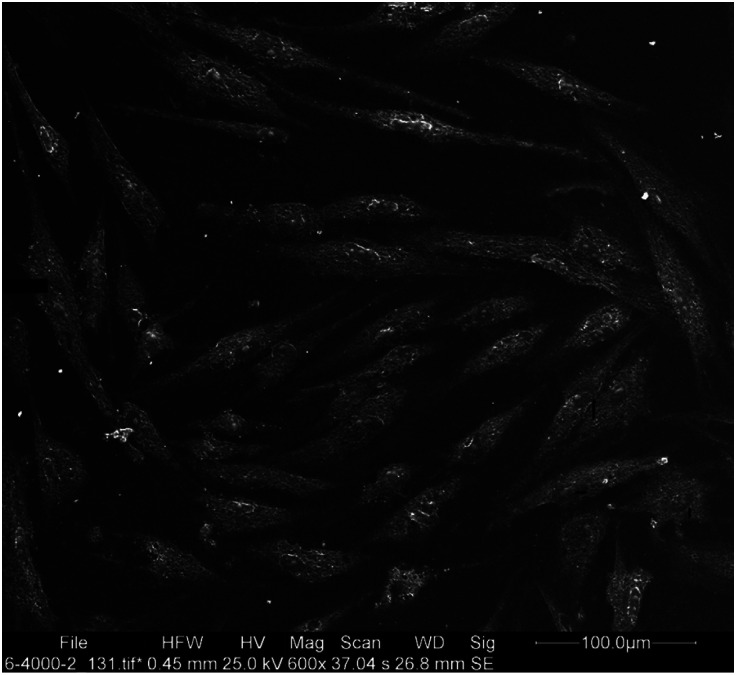
SEM micrograph of the control group.

**Figure 2:**
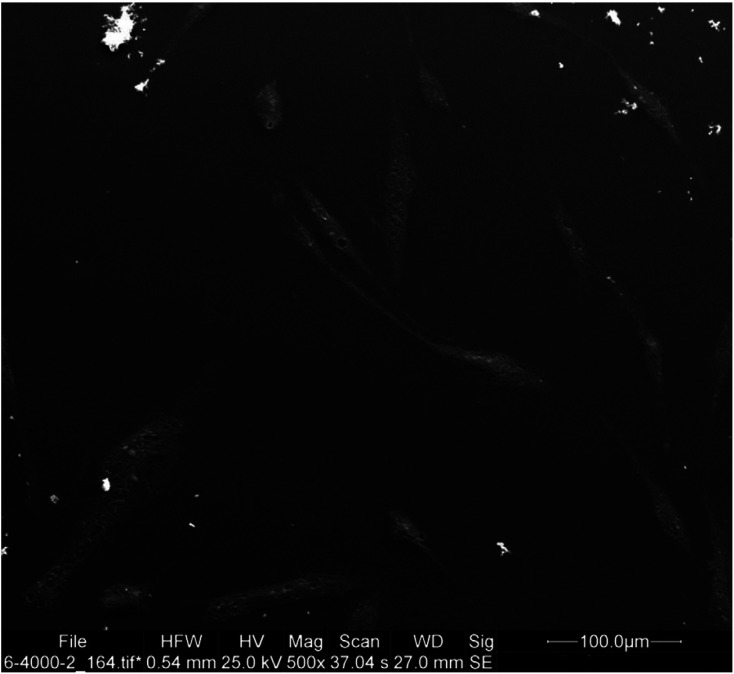
SEM micrograph of the MTAD group showing the lowest number of flat cells.

The fixed specimens were dehydrated by immersion in graded concentrations of ethanol and water. After the final passage in pure ethanol, the dehydration process was accomplished by immersion in hexamethyl disilazene for 30 minutes. The dehydrated cells were gold sputter coated and underwent SEM assessment as explained by Trylovich *et al*. (17). Three SEM micrographs were obtained as representatives of the entire surface. The photomicrographs were obtained with +15-degree angle from three areas with no overlap with each other along a hypothetical diametrical line. The cells were counted and the mean, minimum, and maximum number of cells were reported (Figs. 1,2).

-*Statistical analysis*:

The five groups were compared using the one-way Kruskal-Wallis test.

## Results

**Figure 3:**
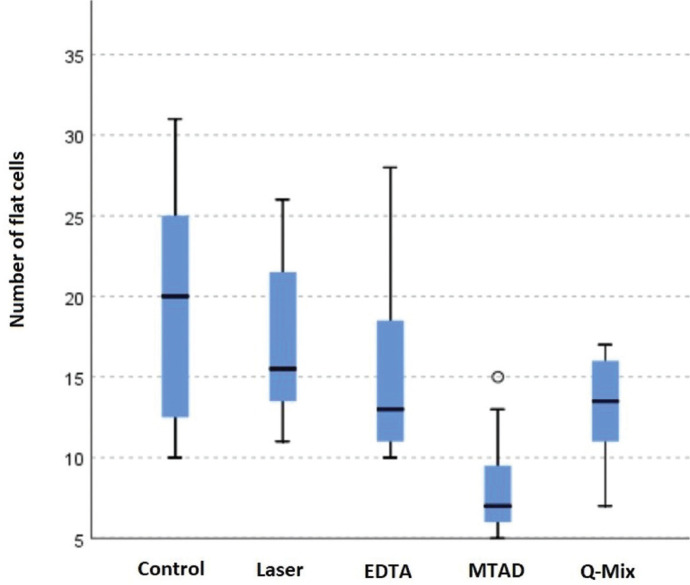
Comparison of the number of flat cells in the experimental groups using independent-samples Kruskal-Wallis test.

SEM assessment revealed that the cells were round or flat in shape. Cells with firm attachments (due to the presence of pseudopod-like processes) were flat; while, weakly attached cells with limited pseudopod-like processes were round in shape. The specimens in the MTAD group showed the minimum number of flat cells with a mean value of 8 cells attached to each specimen. Although the control, laser, EDTA and QMix groups had the highest number of flat cells in an orderly manner, the difference among them was not significant in this respect (*P*>0.05; Fig. 3).

## Discussion

Root canal disinfection is the first step in any regenerative process. The second step involves the transfer of the SCAPs from the apical papilla into the root canal after induction of bleeding (2). Type of dentin pretreatment after root canal disinfection and prior to bleeding induction can significantly affect the behavior of stem cells in terms of migration, attachment, and differentiation. In order to achieve successful regeneration, SCAPs should necessarily adhere to the intracanal dentin after migration. Thus, an ideal irrigant or laser type should be biocompatible, effectively disinfect the root canal, and induce the adhesion of SCAPs to dentin (9).

This study compared the effects of dentin conditioning with EDTA, MTAD, QMix and Er:YAG laser on the attachment of SCAPs. The results indicated that application of different irrigants and Er:YAG laser irradiation changed the behavior of SCAPs. Irrigation with MTAD significantly inhibited cell attachment while the other three modalities induced cell attachment.

According to the literature, MTAD has lower cytotoxicity than 17% EDTA, 5.25% NaOCl, and QMix; in other words, it reportedly has a higher biocompatibility than the conventional irrigants (11,18,19). Also, it is an effective disinfectant that optimally eliminates the smear layer (20,2). However, despite the abovementioned advantages, it resulted in minimum cell adhesion to dentin in the present study. Studies focusing on stem cell attachment to dentin are very limited. However, in line with the present results, Ring *et al*. (1) found that surfaces irrigated with sodium hypochlorite and MTAD showed minimal cell adhesion. Although the cytotoxicity of NaOCl in combination with MTAD was slightly lower than that of NaOCl alone, and NaOCl plus EDTA, it appears that lower biocompatibility of MTAD in their study was due to its application along with NaOCl, which always has a higher cytotoxicity and lower biocompatibility than routine endodontic irrigants. Ghandi *et al*. (21) indicated that irrigation with MTAD had no advantage over saline, and did not induce fibroblast adhesion. According to the results of previous studies and the current findings, MTAD should not be selected as an irrigant for the revascularization procedures because it has no confirmed positive effect on attachment of SCAPs to dentin.

The chelating agents such as EDTA eliminate the mineral part of the smear layer and create a clean dentin surface with open dentinal tubules, which is an ideal substrate for the attachment of SCAPs.

EDTA is extensively used after root canal disinfection with NaOCl, and significantly supports the viability, attachment, and differentiation of stem cells. In fact, EDTA is capable of reversing the adverse effects of NaOCl (22). It has been reported that dentin conditioning with EDTA can significantly enhance the close contact of cells with dentin (1,9,15,22,23). This valuable property was also confirmed in the present study, and is probably related to the exposure of dentin extracellular matrix components such as glycosaminoglycans and types I, III and V collagen, which are all critical for the attachment of SCAPs (22).

In this study, the specimens of MTAD and QMix groups irrigated for 5 min, but irrigation time for EDTA group was 1 min. As De-Deus *et al*. found, dentin microhardness significantly reduced with increasing time of application of EDTA more than 1 min(24). Beside this, other studies have shown similar effect of using EDTA for 1,3 or 5 min on elimination of smear layer (25,26).

Another study indicated that negative effect of EDTA on cell viability increased with increasing application time(27). Whereas the optimal irrigant for immature roots must be the one with the lowest adverse effect on mechanical properties of the thin dentin , and the stem cells viability, and at the same time, desired properties for the irrigant in clinical setting such as smear layer removal and releasing growth factors must be preserved, we considered 1 min application of EDTA group as an optimal irrigating time, versus 5 min for MTAD and QMix groups.

Er:YAG laser, which has recently gained increasing popularity among the clinicians, has a wavelength of 2.94 µm, which is in the near-infrared range, and is well absorbed by water. Thus, it does not increase the temperature of the target tissue following irradiation. Lasers are extensively used for root canal disinfection in endodontic treatments. Naghsh *et al*., (28) reported that Er:YAG laser had a superior performance to CO2 laser with regard to induction of fibrin clot formation and attachment of blood cells. Their results were in agreement with the present findings and previous studies on this topic (29,30). Er:YAG laser irradiation creates an ideal surface for cell attachment. It eliminates the mineralized tissues and creates porosities on the dentin surface, which would result in extensive exposure of dentin collagen. Exposure of collagen fibers enhances the process of cell adhesion (28-30).

Divito *et al*. (14) demonstrated that application of Er:YAG laser after irrigation with EDTA resulted in a cleaner surface and exposure of higher number of open dentinal tubules compared with the application of EDTA alone. Bolortuya *et al*. (31) assessed the adhesion of fibroblasts to dentin surfaces irradiated by Er:YAG laser and reported a significant increase in cell attachment following laser therapy compared with the application of RC-Prep.

In addition to antimicrobial properties, QMix can effectively eliminate the smear layer. Evidence shows that QMix is more biocompatible than EDTA, NaOCl, and chlorhexidine (6-8). According to a histological study, application of QMix resulted in smaller number of inflammatory cells compared with EDTA (6). Eliot *et al*. (5) showed that QMix was superior to EDTA in terms of smear layer removal and exposure of dentinal tubules. Another study also revealed the superiority of QMix to MTAD with regard to smear layer removal (32). A recent study found no significant difference among QMix, MTAD, and EDTA regarding smear layer removal from the middle and coronal thirds of the root; however, QMix had a better performance than EDTA in the apical third in this respect (33).

To the best of the authors’ knowledge, no study has been conducted on cell adhesion to dentin following irrigation with QMix. However, the results of the available studies regarding the cytotoxicity of QMix, EDTA, and MTAD are controversial. In the present study, QMix was compared with other commonly used irrigants, and the results indicated that QMix is a promising irrigant and can contribute to a successful outcome in regenerative treatments.

The present study indicated that although smear layer removal capability, biocompatibility, and antimicrobial activity are ideal properties required for dentin conditioning in a successful regenerative process, these properties are not enough since MTAD is an antimicrobial chelator with optimal biocompatibility; however, it resulted in minimum cell adhesion in the present study and also the limited studies available on this topic (1,21). Irrigants or lasers used for tissue engineering should be able to induce the migration, attachment, and differentiation of cells. To date, the available literature indicates that MTAD (unlike other irrigants evaluated in this study) is not suiTable for regenerative procedures especially when reports regarding successful treatment with other cheaper and more easily available irrigants are present.
